# Microfluidics as a Novel Tool for Biological and Toxicological Assays in Drug Discovery Processes: Focus on Microchip Electrophoresis

**DOI:** 10.3390/mi11060593

**Published:** 2020-06-15

**Authors:** Giuseppe Caruso, Nicolò Musso, Margherita Grasso, Angelita Costantino, Giuseppe Lazzarino, Fabio Tascedda, Massimo Gulisano, Susan M. Lunte, Filippo Caraci

**Affiliations:** 1 Oasi Research Institute—IRCCS, 94018 Troina (EN), Italy; grassomargherita940@gmail.com (M.G.); carafil@hotmail.com (F.C.); 2 Department of Biomedical and Biotechnological Sciences (BIOMETEC), University of Catania, 95125 Catania, Italy; nmusso@unict.it (N.M.); lazzarig@unict.it (G.L.); 3 Department of Drug Sciences, University of Catania, 95125 Catania, Italy; angelita25costantino@gmail.com (A.C.); m.gulisano@unict.it (M.G.); 4 Department of Life Sciences, University of Modena and Reggio Emilia, 41125 Modena, Italy; tascedda@unimore.it; 5 Center for Neuroscience and Neurotechnology, University of Modena and Reggio Emilia, 41125 Modena, Italy; 6 Molecular Preclinical and Translational Imaging Research Centre-IMPRonTE, University of Catania, 95125 Catania, Italy; 7 Interuniversity Consortium for Biotechnology, Area di Ricerca, Padriciano, 34149 Trieste, Italy; 8 Ralph N. Adams Institute for Bioanalytical Chemistry, University of Kansas, Lawrence, KS 66047-1620, USA; slunte@ku.edu; 9 Department of Pharmaceutical Chemistry, University of Kansas, Lawrence, KS 66047-1620, USA; 10 Department of Chemistry, University of Kansas, Lawrence, KS 66047-1620, USA

**Keywords:** toxicology, drug screening, microchip electrophoresis, carnosine, organs-on-a-chip, organoids, 3D bioprinting

## Abstract

The last decades of biological, toxicological, and pharmacological research have deeply changed the way researchers select the most appropriate ‘pre-clinical model’. The absence of relevant animal models for many human diseases, as well as the inaccurate prognosis coming from ‘conventional’ pre-clinical models, are among the major reasons of the failures observed in clinical trials. This evidence has pushed several research groups to move more often from a classic cellular or animal modeling approach to an alternative and broader vision that includes the involvement of microfluidic-based technologies. The use of microfluidic devices offers several benefits including fast analysis times, high sensitivity and reproducibility, the ability to quantitate multiple chemical species, and the simulation of cellular response mimicking the closest human in vivo milieu. Therefore, they represent a useful way to study drug–organ interactions and related safety and toxicity, and to model organ development and various pathologies ‘in a dish’. The present review will address the applicability of microfluidic-based technologies in different systems (2D and 3D). We will focus our attention on applications of microchip electrophoresis (ME) to biological and toxicological studies as well as in drug discovery and development processes. These include high-throughput single-cell gene expression profiling, simultaneous determination of antioxidants and reactive oxygen and nitrogen species, DNA analysis, and sensitive determination of neurotransmitters in biological fluids. We will discuss new data obtained by ME coupled to laser-induced fluorescence (ME-LIF) and electrochemical detection (ME-EC) regarding the production and degradation of nitric oxide, a fundamental signaling molecule regulating virtually every critical cellular function. Finally, the integration of microfluidics with recent innovative technologies—such as organoids, organ-on-chip, and 3D printing—for the design of new in vitro experimental devices will be presented with a specific attention to drug development applications. This ‘composite’ review highlights the potential impact of 2D and 3D microfluidic systems as a fast, inexpensive, and highly sensitive tool for high-throughput drug screening and preclinical toxicological studies.

## 1. Introduction

As it has been described about 15 years ago by Whitesides, “microfluidic is the science and technology of systems that process or manipulate small (10^−9^ to 10^−18^ L) amounts of fluids, using channels with dimensions of tens to hundreds of micrometres” [1]. The development of the first microchip instrument goes back to 1979, when Terry et al. described a miniature gas analysis system fabricated out of silicon [2]. Approximately 10 years later, Manz and co-workers published a paper describing the use of micromachining to build up a miniaturized total chemical analysis system, laying the foundations for the development of an integrated microfluidic system using capillary electrophoresis (CE) [3]. Since then, the number and types of microfluidic-based technologies have dramatically increased and they have been applied in a number of fields including chemistry, biochemistry, physics, biology, and molecular biology [4,5,6,7]. The increased interest of the research community for these technologies is supported by thousands of publications in peer-reviewed journals and conference proceedings [8].

Microfluidic devices offer many advantages for the analysis of the content of single or multiple cells compared to standard methods (e.g., flow cytometry, cell imaging, liquid chromatography, and CE). Representative applications include the simulation of cellular responses that mimic the in vivo environment, such as relaxed or constricted blood vessels, the ability to grow and manipulate a single or group of cells within a specific compartment of the chip, and the possibility to integrate on-line sample preparation and subsequent analysis [9,10,11,12,13,14]. Furthermore, this technology allows for the study of complex systems by reproducing the realistic micro-anatomy and activities, mechanics, and physiological response of specific organs (organs- and organoids-on-a-chip), therefore allowing the shift from flat two-dimensional (2D) cell-based systems to more complex and sensible three-dimensional (3D) architectures [15,16]. Organoids are 3D cellular clusters derived from stem cells or progenitor cells that self-organize in an artificial extracellular matrix and are spatially and dynamically similar to their in vivo counterparts [17]. As a part of the organoid, stem cells and differentiated components are strictly regulated and are able to interact with the surrounding microenvironment provided by the culture medium [17]. The use of organoids is emerging as a promising technology to mimic accurately both human physiology and human pathologies [18], with interesting opportunities for drug discovery and preclinical toxicology [19].

Among the microfluidic-based technologies, microchip electrophoresis (ME)—the miniaturized form of conventional CE—has grown to be one of the most widely used methods. ME can be used to separate and detect multiple analytes from a single sample, as well isolate the molecule of interest from interferences. The very short analysis times characteristic of ME (less than a minute) make it useful for high throughput single-cell analysis and the detection of chemically labile species. Lastly, it is compatible with a number of detection platforms, including fluorescence, electrochemical, and mass spectrometric detection [20]. Additionally, the introduction of the sample—as well as its manipulation, reaction, and detection—can all be performed ‘on-chip’ in a very small volume (from micro- to few nanoliters). The fast separations make it possible to analyze samples sequentially, with minimal time delay between injections and to monitor short-lived species—such as nitric oxide (NO) [21], superoxide (O_2_^−•^) [22], and peroxynitrite [23]—the balance of which is often implicated in the pathophysiology of several diseases (e.g., oxidative stress-driven pathologies). Lastly, ME represents an ideal system to study the behavior of a specific cell population by allowing the biochemical characterization of the content of individual cells [13,24,25,26,27] and can be used to investigate individual differences in cellular response due to the same or different stimuli [28,29].

In the present review we will discuss the application of microfluidic-based technologies to drug discovery and development as well as toxicological studies. Systems of different levels of complexity (from 2D to 3D cell systems) will be described, with attention focused on the multiple utilizations of ME in this field. We also provide new unpublished ME results showing the applicability of ME coupled to laser-induced fluorescence (ME-LIF) and electrochemical detection (ME-EC) to study inducible nitric oxide synthase (iNOS) activity in macrophages and NO degradation in a cell-free system, respectively.

## 2. Microchip Electrophoresis: Technical Characteristics and Basic Principles

To fully understand ME, it is important to know the basic principles of its “non-miniaturized” form, CE, an analytical technique in which the separation of ions occurs based on the electrophoretic mobility following the application of a voltage across a buffer filled fused silica capillary [30]. The key factors determining the electrophoretic mobility in a given media are the charge and hydrodynamic radius of the molecule. The velocity of charged species (ion) toward the electrode of opposite charge is directly proportional to the applied electric field. Therefore, faster separations can be achieved at higher field strengths. If two species have the same hydrodynamic radius, the one with the higher charge will have a greater electrophoretic mobility and if two molecules have the same charge, the species having a smaller hydrodynamic radius will have a greater electrophoretic mobility. Molecules migrating toward the anode have a negative electrophoretic mobility, while those moving toward the cathode have a positive electrophoretic mobility. Neutral compounds have an electrophoretic mobility of zero since they are not attracted to either electrode.

With reference to separations taking place in substrates that have charged surfaces—such as silica, glass, or polydimethylsiloxane (PDMS)—another factor to be taken into account is the electroosmotic flow (EOF). EOF is the movement of bulk liquid caused by the electrical double layer generated at the charged capillary wall by adjacent solvent ions. When a voltage is applied, hydrated solvent ions (in normal polarity, these would be cations) migrate toward the electrode of opposite charge (cathode), dragging the solvent with them. If the EOF is strong enough, it will cause the bulk movement of all species towards the cathode. Therefore, if the detector is placed at the cathode, the migration order will be cations, neutrals, and then anions [31]. The magnitude of the EOF is a function of the zeta potential set up at the capillary wall. This can be controlled by the modulation of an external voltage, chemical modification of the capillary surface, and changes in background electrolyte composition such pH and ionic strength [32].

**Figure 1:**
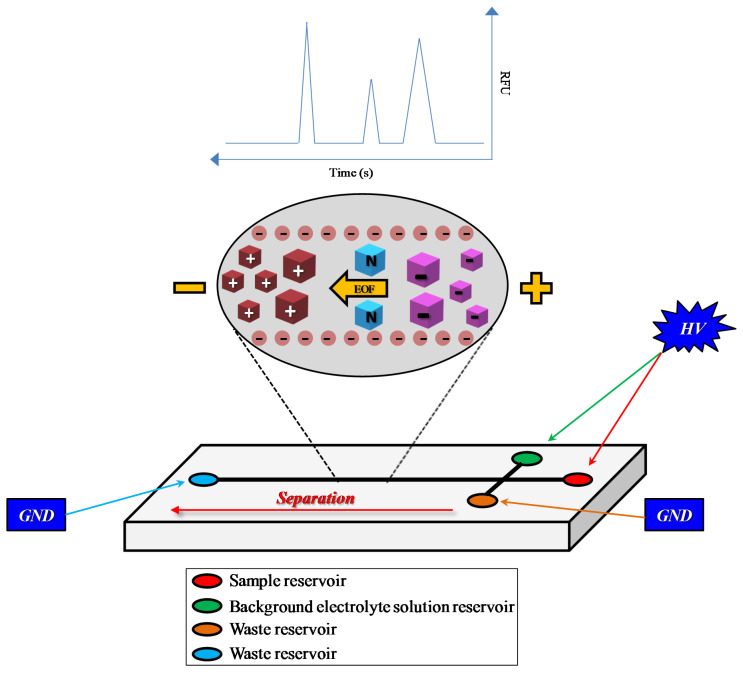
Typical microchip design (simple ‘T’ design) consisting of a separation channel and two side arms. EOF = electroosmotic flow; GND = ground; HV = high voltage.

With regards to ME, the separation process occurring on the chip is based on the same principle as with CE, but the microfluidic separation device has unique features coming from the smaller planar format [20,33]. The typical ME design is a simple ‘T’ consisting of a separation channel and two side arms [34]. Fluid reservoirs (for a total of four) are positioned at the ends of each channel: one is used for sample introduction, another one is needed for the background electrolyte solution, while the other two act as waste reservoirs (Figure 1).

The application of a high voltage to each reservoir is achieved by the presence of platinum electrodes directly connected to power supplies, while a persistent buffer flow is directed towards both sample and waste reservoirs [35]. For ME, the use of many different substrates have been employed with glass, plastic, and PDMS representing the most widely used due to their excellent electroosmotic and electrokinetic properties [5,36,37,38]. In addition, these materials are all optically transparent and devices can be fabricated using standard photolithographic techniques. Glass has the advantage that it is similar in composition to fused silica so it is easy to transfer methods from CE to ME. Surface adsorption of proteins is a common drawback observed using these materials, and can be reduced by incorporating non-ionic surfactants or bovine serum albumin into the run buffer. Another common approach is to chemically modify the channel surface to reduce interaction of proteins with the wall. For two relatively recent reviews that address the role of protein adsorption in ME and CE separations, see [39,40]. 

Four different types of injection approaches have been employed for ME experiments. These are pinched [41], floating [42], gated [43], and dynamic [44]. After the injection of the sample is realized through the intersection of the microchannels (integrated injector), the analytes are separated based on their electrophoretic mobilities (and EOF if present) and then detection is performed. The latter can be achieved by one of the following: (1) chemiluminescence (ME-CL); (2) laser-induced fluorescence (ME-LIF); (3) electrochemical detection (ME-EC) (including conductivity); (4) mass spectrometry (ME-MS); (5) UV/Vis (ME-UV/Vis). Among them, because of its low detection sensitivity, UV/Vis absorption is the least used one, while LIF represents the most widely applied detection method, probably because of its high detection sensitivity due to a low background signal [20].

As will be described in more details in the following sub-sections, ME covers a wide range of applications including biology, chemistry, engineering, toxicology, drug screening, and medicine [45,46]. ME has several advantages over more conventional methods for these types of studies. The primary advantage is the ability to separate and detect several different analytes in a single small volume sample. For example, this approach makes it possible to detect a drug and its metabolites, separate several DNA strands, identify multiple proteins in a clinical sample, and analyze several reactive oxygen and nitrogen species in a cell. In addition, the analysis time is generally much shorter than more conventional separation methods, allowing for the detection of short-lived chemical species. For both ME and CE, very small sample volumes are needed so it is possible to analyze individual cells or make multiple measurements from a single precious sample. The planar format and the use of microfluidics to move fluids make it possible to integrate other functions, such as sample preparation, on the same chip. The chips can also be mass produced and be disposable, which is good for clinical applications to avoid cross contamination. With regards to ME coupled to the use of fluorescent probes (ME-LIF), such as 4-amino-5-methylamino-2′,7′-difluorofluorescein diacetate (DAF-FMDA) and MitoSOX Red, it is worth noting that ME allows to detect the ‘real’ fluorescence coming from the reaction between the probes and the molecules of interest, and to discriminate the considered compounds from other side products generating fluorescence [47].

Among the disadvantages of ME we should consider that it is a serial analysis method so only one sample is run at a time. Therefore, even with subminute separations, it is not as high throughput as microtiter plate-based assays that can run hundreds of samples at a time. It is also less easy to automate than CE or high-performance liquid chromatography (HPLC). The small volume requirements can also be a disadvantage if one is not sample limited and needs preconcentration to achieve the desired limits of detection (LOD). Another potential drawback is passivation of the channel surface causing a change in migration times for analytes if the chip is used for multiple runs. For a more exhaustive discussion of advantages and disadvantages of ME (and CE) compared to conventional methods also refer to Shilly et al. [48] and Breadmore [49].

### 2.1. Role of ME in Drug Discovery and Development and in Preclinical Toxicology

**Figure 2:**
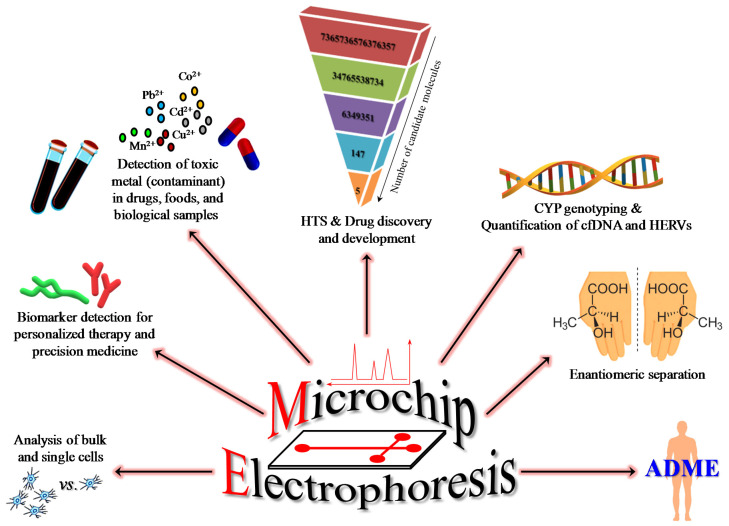
Broad applications of microchip electrophoresis. HTS = high throughput screening; ADME = absorption, distribution, metabolism, and excretion; cfDNA = circulating cell-free DNA; HERVs = human endogenous retroviruses.

ME, as well as its ‘precursor’ CE, are considered promising tools in the pharmaceutical field, with specific regard to the characterization of pharmaceutical components and drug discovery [50,51]. More recently developed and promising ME-based methods have significantly broadened the application of this method to include genotyping, detection of contaminant in drugs, foods, and biological samples as well as the analysis of samples relating to forensic and clinical toxicology [46] (Figure 2).

ME has become a powerful tool for analysis of drugs and toxic compounds in different matrices, increasing the understanding of the pharmacodynamic and pharmacokinetic profile of drugs under development as well as the prediction of toxicity of these compounds [45]. This information is extremely important for the determination of the appropriate dose to reduce the risk of the development of side effects, resulting in better treatment protocols. For example, a two-electrode electrochemiluminescence detection method for ME was developed by Pan et al. [52]. Their system was able to separate and detect atropine, anisodamine, and proline with a LOD of 1 µM. Zhang and colleagues, by employing a compact and low-cost ME device coupled to light-emitting diode-induced fluorescence, determined the presence of sulfonamides (sulfadiazine, sulfamethazine, and sulfaguanidine) in pharmaceutical formulations and rabbit plasma [53], showing the potential of their method to study the pharmacokinetics of drugs.

During the drug development process, a key role is played by metabolic profiling, including identification and determination of the toxicity of metabolites. Therefore, a selective and sensitive ME method that allows for the simultaneous determination of all possible metabolites could represent a relevant step to improve drug discovery processes. In this regard, Nordman et al. carried out a rapid and sensitive drug metabolism study employing a ME-electrospray ionization (ESI)/MS system for the identification and separation of paracetamol and tramadol metabolites in biological samples (urine) collected from healthy volunteers after drug (500 mg of paracetamol or 50 mg of tramadol) administration [54]. A total of 10 metabolites (6 of tramadol and 4 of paracetamol) were detected and quickly (30–35 s) separated from each other and detected by MS using this approach.

During the drug development process, the selection of the ‘right’ enantiomer to enhance clinical efficacy or avoid drug toxicity is of the utmost importance [55]. The separation of D-penicillamine from its highly toxic L-enantiomer was performed by Yang and co-workers [56]. Both enantiomers were derivatized with fluorescein isothiocyanate and detected following electrophoretic separation by light emitting diode induced fluorescence detection. Their approach can be interesting for researchers involved in drug screening since the background electrolyte can be enriched with chiral selectors (e.g., carboxymethyl-β-cyclodextrin) that make it possible to allow multiple runs in a very short span of time, as well as provide better separation/identification of the analytes.

As recently highlighted by Egorova and Ananikov, transition elements (groups 3 through 12 of the periodic table) can lead to toxic effects as a consequence of their easy change in oxidation state and their interaction with charged molecules [57]; nevertheless, it is important to mention that transition metal complexes also represent potential therapeutic agents for the treatments of different human disease such as inflammatory disorders [58]. The toxicity of a metal depends on several factors including the oxidation state and ligands that are used. Therefore, it is important to identify and separate a specific metal or metal complex to clarify its behavior, with regard to the conditions and the environment in which it was found. The field of metal analysis by ME began more than 20 years ago [59]. Since then, encouraging progress in this area has been made in exploring rapid metal complex speciation in addition to their activity, toxicity, and potential side effects [60,61,62]. Liu et al. developed a ME coupled with contactless conductivity detection device for the separation and detection of heavy metal ions (Mn^2+^, Pb^2+^, Cd^2+^, Co^2+^, and Cu^2+^) within 100 s [63]. In a different study, Deng and Collins used a nonaqueous background electrolyte for the separation of toxic metal ions by ME [64]. The combination in this system of borofloat glass microchips with silica microcolumns (C18), along with a sample pre-concentration step, dramatically improved the LODs of the above-mentioned metal ions, going from a 311-folds improvement for Cd^2+^ to a 730-folds improvement observed for Pb^2+^. It is then clear how ME provides the opportunity for the precise and fast separation and identification of a specific toxic metal in sample mixtures.

Pharmaceutical preparations frequently contain a wide range of excipients, each with a defined pharmaceutical purpose [65]. In this regard, Troška et al. described a ME method with contact conductivity detection for simultaneous determination of three frequently used pharmaceutical excipients (methylparaben, propylparaben, and erythrosine), with a LOD ranging from 0.5 to 11.1 μmol/L [66]. Their method was also applied to the analysis of pharmaceutical syrups, giving recoveries of the analytes in the range of 86.1–96.6%.

Several ME systems have been commercialized for drug screening purpose. One of the most popular system belonging to this category is represented by the LabChip instrument from PerkinElmer, which uses a vacuum system to move a numerous samples from a multiwell plate into the microfluidic separation channel with fluorescence detection (www.perkinelmer.com). This high-throughput screening (HTS) technology was used by Perrin et al. to screen 32000 compounds, leading to the identification of four novel chemical series of serine/threonine kinase inhibitors [67]. During the last 15 years, this system has been used for different targets including enzyme classes and protein–RNA interactions [51,68,69,70,71,72,73]. By using a different commercial ME system (Shimadzu) (www.shimadzu.com), Ludwig and colleagues demonstrated that the chiral separation of 19 drugs could be achieved in less than 1 min with high reproducibility, with the fastest separation obtained in only 2.5 s [74]. One of the issues with therapeutics circulating in blood is the possible degradation by enzymes and serum components. This makes it hard to predict how, and in which manner, each molecule of interest will tolerate the in vivo environment as a consequence [75]. Piparia et al. described the use of a combined CE-microfluidic device system obtained by synergizing a CE-based microfluidic device, commercially known as LabChip GXII, with a fluorescent labeling method (Pico Protein probe) to investigate the pharmacokinetic parameters of human monoclonal antibodies, directly from biological serum samples [76].

In addition to the ones mentioned above, there are many commercial vendors for microfluidic chips and systems that can be used for ME. These include ChipShop (www.microfluidic-chipshop.com), Labsmith (labsmith.com), A-Line (alineinc.com), MiCrux (www.micruxfluidic.com), Micronit (www.micronit.com), Agilent (www.agilent.com), and 908 devices (908devices.com) to name a few.

From all the above we can conclude that ME methods represent a powerful tool for the analysis of small sample volumes yielding fast separations of drugs and metabolites for the determination of the toxicity profile of a molecule before its selection as a candidate drug to progress to clinical trials.

### 2.2. ME for the Analysis of Bulk and Single Cells

Over the past two decades, the development of a plethora of ME-based systems to study biological events as well as toxicity and drug screening in bulk (groups of cells) and in single cells has been described.

PC-12 cells are often used as model neurons for in vitro studies of neurotransmitters. Shi et al. described a ME system composed by an inverted fluorescence microscope provided of a high-pressure mercury lamp as excitation source and a photon counter as the detector allowing the determination of neurotransmitters in PC-12 cells [77]. The ME-fluorescence determination of seven derivatized catecholamines and amino acids was successfully performed with a LODs ranging from 0.13 to 0.85 fmol. The same research group used two different derivatizing agents, fluorescein-isothiocyanate (FITC) and ortho-phthalaldehyde (OPA), for the analysis of amino acids in human vascular endothelial cells (ECV-304) [78]. During the last 10 years, a considerable contribution to the development of ME-based methods suitable for separation, detection, and quantification of reactive oxygen species (ROS), reactive nitrogen species (RNS), and antioxidants in immune cells (especially macrophages) as well as for the near real-time monitoring of drugs and neurotransmitters in vitro and in vivo has been given by Lunte’s group [79]. It is well-known that NO and O_2_^−•^ play critical roles in many physiological and pathological processes [80,81]. A ME-LIF method was developed and used for the measurement of NO in lipopolysaccharides (LPS)-stimulated T-lymphocytes (Jurkat cells – Clone E6) [82,83] and O_2_^−•^ in phorbol 12-myristate 13-acetate (PMA)-stimulated macrophages (RAW 264.7) [10], by employing DAF-FMDA and MitoSOX Red probes, respectively. The reaction between O_2_^−•^ and NO, which leads to the formation of peroxynitrite, is very fast [84]. This makes the simultaneous detection of these reactive species very difficult to perform. In this regard, a ME-LIF method for the simultaneous detection of these species was developed that allowed for the determination of the ratio of NO to O_2_^−•^ produced in macrophages under physiological and pro-inflammatory conditions [21]. Gunasekara et al. established a ME-EC method for profiling cellular nitrosative stress markers, including the major oxidative metabolite of NO, nitrite (NO_2_^−^) [85]. In the same paper, a comparison of the results achieved with ME-EC was made with the widely used Griess assay for NO_2_^−^ measurement with good agreement. Very recently, Siegel et al. developed an optimized method using ME-EC for the separation and detection of NO_2_^−^ in bulk cell lysates [38]. The use of a platinum black working electrode resulted in a slight signal enhancement for NO_2_^−^ (2-fold) as well as for ascorbic acid and hydrogen peroxide compared to the conventional set-up.

The application of ME-based technologies has led to a better understanding of the intriguing effects of carnosine (beta-alanyl-L-histidine), a peptide with a high therapeutic potential, on immune cells under physiological and pathological conditions. Specifically, it was demonstrated that carnosine is able to downregulate the intracellular concentration of NO and O_2_^−•^ in both macrophages [22,86] and microglial cells [47]. In a separate study carried out by Fresta et al., the physiological concentration of carnosine present for macrophages as well as the enhanced uptake of this molecule made by stimulated macrophages were determined [5]. Lastly, the protective effects of carnosine in counteracting the total ROS production induced by carbon nanoparticles in murine microglia (BV-2) and human alveolar basal epithelial cells (A549) was demonstrated [34].

One of the objectives of biology is to understand the mechanisms operating in a single cell, the minimal functional unit of life. Cells within the same population are not identical and do not necessarily behave in the same manner due to this heterogeneity. In fact, during stress or perturbation, some sub-populations adapt better to the new microenvironment, resulting in higher survival or even growth rates, making the base for the subsequent main population [87]. A system allowing the electrophoretic separation of Oregon green, calcein AM, and carboxyfluorescein dyes released from single unstimulated leukocytes cells was reported almost two decades ago by McClain et al. [88]. The measured cell analysis rates obtained by employing this method were reported to be from 100 to 1000 times faster than those observed when using conventional CE. The tripeptide γ-l-glutamyl-l-cysteinyl-glycine, better known as glutathione (GSH), represents one of the most important antioxidant compounds produced by cells [89]. The rapid quantification (fmol levels) of this antioxidant in single rat liver cells was recently achieved by Sin et al. through the use of ME coupled to a chemiluminescence detection system [90]. The detection and quantification of GSH along with total ROS was achieved in single erythrocytes using 2,3-naphthalene-dicarboxaldehyde and dihydrorhodamine 123, respectively, by Ling, Yin, and Fang [91]. This method allowed not only the simultaneous determination of GSH and ROS, but also made it possible to monitor variations in the cellular content of these species in response to external stimuli. The LOD was 0.5 amol for ROS and 6.9 amol for GSH, with an average cell throughput of 25 cells/hour. Several years later, Metto et al. reported a high throughput ME device allowing the analysis of 200 individual leukocytes cells (Jurkat cells – Clone E6) in only 20 min [9]. The system was employed to measure the NO produced by these cells under physiological and pro-inflammatory (LPS stimulation) conditions, showing a 2-fold increase in NO production in LPS-stimulated cells. A ME-MS platform with double cell lysis nanoelectrodes for automated single cell (PC-12) analysis has also been described by Li and colleagues [92]. Intracellular levels of dopamine (DA) and glutamic acid (Glu) were determined for a large number of individual intact PC-12 neuronal cells and compared to those exposed to potassium chloride (KCl). Their results showed that the concentration of DA was higher than Glu in the KCl stimulated cells, and both varied from cell to cell. A ME-LIF method was developed to simultaneously quantify O_2_^−•^ and NO at the single-cell level in the same cell type by using a consecutive gated injection-based microfluidic device [93]. The authors were able to monitor the production of both species before and after stimulation with 6-hydroxydopamine (6-OHDA), a neurotoxin widely used to induce models of Parkinson’s disease (PD). They also showed the protective effect exerted by green tea polyphenols in downregulating O_2_^−•^ and NO generation in single 6-OHDA-stimulated PC-12 cells.

### 2.3. ME for Biomarker Detection in Personalized Therapy and Precision Medicine

ME represents an analytical method for the rapid determination and accurate quantification of components belonging to one of the four major classes of biological macromolecules (DNA, carbohydrates, lipids, and proteins) in clinical samples, representing a diagnostic and prognostic tool for the early diagnosis and better characterization of several diseases.

In 2012, the usefulness of ME for genotyping assays was evaluated by Mizukami et al. for the detection of detecting the four-base pair deletion of the canine multidrug-resistance (MDR1) gene [94]. Compared to the conventional polymerase chain reaction (PCR) method, ME more clearly and rapidly separated the 60-bp (wild-type) and 56-bp (mutant allele) bands resulting in the clear discrimination of the three genotypes MDR1 (+/+), MDR1 (+/−), and MDR1 (−/−). This method also represents a useful tool for human genotyping studies, since at least three single nucleotides polymorphisms (SNPs) were found in the human MDR1 gene [95], which may be related to abnormal sensitivity to multiple drugs. The same year, Poe and colleagues developed a ME method employing a tetraprimer amplification refractory mutation system for the determination of the three biallelic SNPs—CYP2C9*2, CYP2C9*3, and VKORC1 haplotypes A and B—affecting clinical efficacy and toxicity of the oral anticoagulant drug warfarin [96]. The results obtained by analyzing a total of 35 human genomic DNA samples were completely in agreement to those obtained by other conventional and validated PCR methods thus representing a promising tool for a low-cost, reproducible, and rapid platform for genotyping drug clinical efficacy and adverse drug reactions. Very recently, in a study carried out by Fujihara et al., circulating cell-free DNA (cfDNA), that has been directly related to different diseases—such as cancer, diabetes, stroke, inflammation, and myocardial infarction (MI)—was extracted from the plasma of cardiac disease patients and analyzed by ME [97]. The authors demonstrated that fragments of 150–200 bp, 300–400 bp, and 500–600 bp were present in all cardiac patient samples and that a cfDNA ratio of 150–200 bp/500–600 bp was significantly more prevalent in MI patients than in patients with other cardiac diseases, suggesting that this ratio may represent a novel diagnostic biomarker for MI disease. Human endogenous retroviruses (HERVs) are part of the superfamily of transposable and retrotransposable genetic elements and represent almost 10% of the human genome [98]. The abnormal expression of HERVs has been proved for different pathological conditions such as autoimmune, neurodegenerative, and chronic inflammatory diseases [99]. These endogenous retroviruses are often analyzed by using a combination of PCR-based methods and an electrophoretic separation [100,101] and the ‘translation’ from conventional methods to a ME-based technology, as it has been observed for the genotyping studies of MDR1 [94], could represent an alternative strategy for a personalized medicine approach. A study comparing the combination of ME with nucleic acid sequence based amplification, two-step, or one-step real-time PCR to study human papillomavirus (HPV) was achieved by Liu et al. [102]. The coupling of the above-mentioned techniques gave a synergic improvement, shortening the analysis time and improving the sensitivity. Wang, Ni, and Zhou established a ME-based method that was applied to the analysis of the DNA samples obtained from 20 healthy volunteers, allowing the typing of five different SNPs present in the CYP2D6 gene, then showing the feasibility of this method to type several SNPs simultaneously [103]. This can be of relevance to better predict the dosage of antipsychotic drugs, allowing the rapid screening of patients and, thus, a personalized medicine approach, reducing the risk of toxic effects from medication [104]. The same method could help to define the right dose of tacrolimus that is an immunosuppressive drug often administered to patients who have undergone an organ transplant in order to reduce the risk of rejection [105], based on the CYP3A5 genotype in transplant patients [106,107]. The rapid and accurate CYP3A5 and MDR1 genetic polymorphisms analysis could also help to define the dosage of the immunosuppressive drug cyclosporine A, whose pharmacokinetic characteristics are extremely variable among individuals, to be administered during the early stage after renal transplantation [108,109]. In a different study, Minarik et al. described the development of an analytical CE-based method making it possible to easily and rapidly analyzing the resistance to clopidogrel by CYP (2C19/C9) genotyping [110]. SNP genotyping lead to the identification of slow metabolizers characterized by clopidogrel-resistance, representing a cornerstone of cardiovascular treatment in coronary artery disease patients.

Lipids—such as cholesterol, phospholipids, and prostaglandins—can be used as biomarkers [111], although their most popular features are represented by energy storage and formation of cellular membranes. In 2011, Ruecha and colleagues established a system consisting of a PDMS chip, fabricated using standard soft lithography, with amperometric detection at a 25-µm gold wire working electrode which provided the possibility to measure the concentration of cholesterol in bovine serum samples [112]. The authors exploited the enzymatic reaction occurring between the cholesterol (sample solution) and cholesterol oxidase enzyme (buffer solution) producing hydrogen peroxide. The LOD was 38.7 ng/dL (1 nM), well below clinical levels, while the sample throughput was equal to 60 samples/hour. The analysis of lipoproteins, the major extracellular carrier of lipids, is often used as a diagnostic tool, as in the case of atherosclerosis and coronary heart disease. Two low-density lipoprotein sub-classes were separated and identified using a PDMS/glass ME coupled to fluorescence with high speed and high reproducibility [113]. Gilson and Bohn introduced a three-dimensional non-aqueous ME (NAME) system, consisting of two orthogonal microfluidic channels interconnected with each other through a nanocapillary array membrane which ensures fluidic communication, for the electrophoretic separations of fluorescently tagged binary and ternary lipidic mixtures [114]. This is relevant, since the measurement of lipid biomarkers in vivo is made difficult by the low solubility of this molecules in aqueous solution, and the device made it possible to make a correlation between oxidative stress and lipid biomarker levels observed in vivo.

The detection and quantification of proteins is normally performed by expensive and time-consuming methods such as western blot analysis or enzyme-linked immunosorbent assay (ELISA) [115,116,117]. Mohamadi and colleagues performed the ME profiling of fluorescently labeled amyloid-β (Aβ) peptides in the cerebrospinal fluid (CSF) of patients with Alzheimer’s disease (AD) [118]. They first performed the separation and quantification of five synthetic Aβ peptides (Aβ1-37, Aβ1-38, Aβ1-39, Aβ1-40, and Aβ1-42); the same method was then applied for profiling of Aβ peptides (Aβ1-40 and Aβ1-42) in CSF samples from non-AD and AD patients. α-fetoprotein (AFP)-L3 represents a new generation of tumor marker for hepatocellular carcinoma (HCC) [119]. A highly sensitive AFP-L3% (rate of AFP-L3 in total AFP) ME-based assay developed by Kobayashi et al. was demonstrated to be highly sensitive for this marker in HCC patients; by employing this method, pre- and postoperative AFP-L3% were identified as two factors useful to estimate the chance of HCC recurrence after the therapy, the percentage of which increases as the years after treatment increases (ranging from 21.5% at year 1 up to 65.6% at year 5) [120]. More recently, Phillips and Wellner determined the concentration of CCL2, CCL19, CCL21, CXCL8, CXCL12, and CXCL13 chemokines in the CSF samples obtained from preterm infants (severe head trauma or mild forceps trauma during birth) and compared them to those of controls (no pathological or clinical abnormalities) [121]. Their system, consisting of a Micralyne μTK ME device coupled to LIF detection and incorporating replaceable immunoaffinity disks, allowed the complete processing of a sample in 10 min with separation of all analytes (a total of 6) obtained in less than 2 min. Of note, the ME analysis not only identified the groups with mild and severe trauma, but also demonstrated that the severe trauma group could be divided into two sub-groups (good and poor prognosis), correlating with the clinical finding for each patient.

Carbohydrates play a fundamental role in numerous biological processes including cell signaling, cell adhesion, and the regulation of biochemical pathways. Mass spectrometry represents one of the most commonly utilized method for the characterization of carbohydrates [122]. A faster and easier way to analyze carbohydrates in solution, as well as in biological samples, is represented by ME. In this regard, the electrophoretic separation of the N-glycans in blood serum samples obtained by healthy individuals (control) and subjects diagnosed with ovarian cancer prior to and after pharmacological treatment (docetaxel and imatinib mesylate in combination) using ME-LIF was carried out by Mitra et al. [123]. The quantitative intersample differences regarding the N-glycan profile were measured, with analysis times of less than 100 s. Very recently, a method based on the use of a glass ME device with integrated contactless conductivity detector was used to perform the electrophoretic separations of fructose, galactose, glucose, lactose, and sucrose; in order to control the electrolysis process, external reservoirs, connected to the microfluidic platform by a system composed by four saline bridges, were used [124]. The five carbohydrates were separated very quickly (within 180 s) with great efficiency, run-to-run reproducibility, and LOD values ranging from 150 to 740 μmol/L. Maeda and co-workers developed a method using ME with a plastic chip allowing the determination of glucose levels in a complex matrix such as the human blood [125]. In order to achieve this goal, a derivatization of glucose by 2-aminoacridone (AMAC) (90 min) was needed; from the other hand, their system allowed a very low consumption of blood (100 times less) compared to conventional colorimetric analysis. This system represented a highly sensitive and accurate tool for clinical diagnosis in which the determination of blood glucose levels need to be achieved.

### 2.4. ME for Separation and Quantification of Neurotransmitters In Vitro and In Vivo

Continuous monitoring of biomolecules in in vitro as well as in living systems is important for the understanding of neuroinflammation and neurodegeneration phenomena, the bidirectional relationship between a specific drug treatment and the individual variation of drug response, and the evaluation of drug delivery systems [126]. Neurotransmitters are molecules produced by brain cells (i.e., neurons) necessary for the normal functioning of the central nervous system (CNS). These molecules, able to bind to specialized receptors on the target cell, co-ordinate communication between cells and help control cell activity [127]. In this regard, the opportunity to monitor the dynamic changes in neurochemical release, for example after a drug treatment or in the case of a toxic stimulation, can be of great interest.

Li et al. developed a system to study the release of different neurotransmitters in rat pheochromocytoma cell line (PC12) stimulated with a chemical stimulant known to lead to cells depolarization followed by exocytosis, KCl, or alcohol [128]. Their ME-MS system allowed to monitor the DA release from PC-12 cells, which was significantly increased in stimulated cells; the authors were also able to demonstrate an increase in the secretion of serotonin (5-HT) in conjunction with the stop in DA release. This suggests that these two neurotransmitters are packaged into different vesicle pools and are mobilized differently as a consequence of a chemical stimulation. Neurotransmitters have also been measured in animal brain tissues. An example of this type of analysis is given by Vlčková and Schwarz who developed an ME-based method for simultaneous determination of catecholamines (DA, adrenaline, and noradrenaline (NE)) and their *O*-methoxylated metabolites (methoxytyramine, normetanephrine, and metanephrine) in mouse brain homogenates [129]. A simple and rapid method employing an on-line multiple-preconcentration approach combining field-amplified stacking and reversed-field stacking for the simultaneous analysis of 5-HT, NE, and DA neurotransmitters in urine samples by ME-LIF was developed by Zhang et al. [130]. Their optimized system allowed a very quick (3 min) neurotransmitters separation characterized by nanomolar-level LODs (1.69 for 5-HT, 2.35 for NE, and 2.73 for DA).

Many different ME-based methods have also been developed and employed to monitor neurotransmitters in vivo. Wang et al. built up a platform consisting of a dual-chip system (PMDS + glass) coupled to microdialysis sampling to detect rapid neurotransmitters concentration changes during a pharmacological treatment (infusion of L-trans-pyrrolidine-2,4-dicarboxylic acid) in rats [131]. They measured serine (Ser), glycine (Gly), Glu, and aspartate with LODs of 90–180 nM. In a different study Scott et al. described a microdialysis-ME-EC system able to monitor the production of NO_2_^−^ following the subcutaneous perfusion of nitroglycerin, a pro-drug that undergoes complex metabolic biotransformation able to cause vasodilation by NO production [132], in freely roaming sheep [133]. The authors first tested their system in rats to monitor the production of NO_2_^−^ following perfusion of the same pro-drug molecule. Data were collected and analyzed every 60 s for immediate interpretation of concentrations. Sandlin et al. used a ME device with on-line derivatization and separation to monitor Glu levels in brain of rats stimulated with L-*trans*-pyrrolidine-2,4-dicarboxylic acid (PDC) [134]. By using this system—composed of a sample introduction channel, a pre-column for derivatization with OPA, a flow-gated interface, and a separation channel—it was possible to obtain an electropherogram containing a peak corresponding to Glu (derivatized with OPA) every 25 s, demonstrating the ability of this system to monitor dynamic changes in neurotransmitter concentrations in vivo. Very recently, the development of a separation-based sensor for catecholamines based on microdialysis coupled to ME-EC was described by Gunawardhana et al. [135]. The authors first tested their system measuring 3,4-dihydroxy-L-phenylalanine (L-DOPA), 3-O-methyldopa (3-O-MD), homovanillic acid (HVA), 3,4-dihydroxyphenylacetic acid (DOPAC), and DA standards, which were separated in less than 100 s; in a second phase the device was used for monitoring DA release in an anesthetized rat following high K^+^ stimulation.

In the present review, we focused primarily our attention on the use of ME for drug discovery and development and in preclinical toxicology studies, its application for the analysis of bulk and single cells, for the detection of biomarkers in biological fluids, as well as for monitoring neurotransmitters in vitro and in vivo. For further discussion of applications and advances in ME (and CE) see the recent review written by Sibbitts et al. [136] and Ragab and Kimary [137].

We turn in the next sub-section to a new and alternative application of ME to study carnosine effects on NO.

### 2.5. New Application of ME: The Interesting Case of Carnosine and Its Effects on iNOS Activity and on the Degradation of NO

The three different enzymes that make up the nitric oxide synthases family are endothelial nitric oxide synthase (eNOS), neuronal nitric oxide synthase (nNOS), and iNOS and these produce NO through the conversion of L-arginine (Arg) to L-citrulline (Cit) [138]. In contrast to eNOS and nNOS that are constitutively activated, iNOS is strongly expressed in immune cells such as macrophages and microglia and can be induced by pro-oxidant and pro-inflammatory stimuli [47,139], leading to the production of a large amount of NO. Among the natural occurring compounds, the dipeptide carnosine has been shown to be able to decrease the expression of iNOS as well as the concentration of its product, NO, in immune cells [21,47,139].

During the past five years, our research group has been able to show that carnosine: 1) does not inhibit iNOS activity in macrophages cultured under pro-inflammatory conditions [86] and 2) strongly enhances the degradation of NO into its major end-product NO_2_^−^ [86,139]. The following results will highlight how a simple, rapid, and cost-effective ME method (ME-LIF or ME-EC) can be used with or in place of other well-known standard, time consuming, and expensive techniques/methods such as ELISA and HPLC to investigate the effects of carnosine on iNOS activity, as well as on the degradation of NO.

#### 2.5.1. Investigation of the Effects of Carnosine on iNOS Activity in Macrophages by ME-LIF

We have already published data on iNOS activation in macrophages under pro-inflammatory conditions (LPS + IFN-γ). By employing ELISA assay and HPLC with fluorescence detection, we demonstrated that macrophages pre-treated (1 h) with carnosine (20 mM) before stimulation for 24 h with LPS (100 ng/mL) + IFN-γ (600 U/mL) did not show a significant decrease in iNOS protein expression (92.12 ± 6.47%) and activity, measured by the conversion of Arg to Cit, compared with cells stimulated in the absence of carnosine [86].

**Figure 3:**
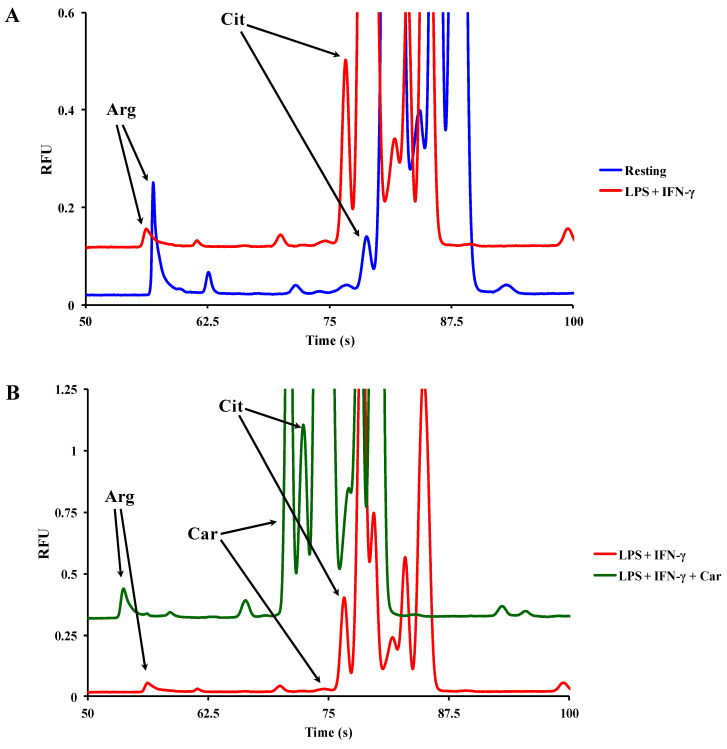
Representative electropherograms of cell lysates showing the change in peak area of Arg, Cit, and carnosine (Car) (**A**) for resting (untreated) macrophages (blue line) and (**B**) for macrophages stimulated with LPS + IFN-γ, in the absence (red line) or in the presence (green line) of carnosine. RFU = relative fluorescent unit.

Here we show unpublished data on the application of ME-LIF to study iNOS activity in macrophages stimulated under the same conditions described above. Glass microfluidic devices fabricated by photolithographic techniques that have been described previously were used to carry out the ME-LIF experiments [140]. Among the four electrokinetic injections mentioned above, we employed a gated injection, occurring for about 500 ms, leading to an injection volume of approximately two picoliters. To perform the gated injection, (1) the high voltage is applied to the sample and buffer reservoirs and the ‘gate’ is established; (2) the buffer voltage floats to 0 and the sample fills the channels; (3) gating conditions are re-established and the sample goes through the separation channel [141]. The determination of the intracellular concentrations of carnosine, Arg, and Cit in murine RAW 264.7 macrophage cell lysates was performed by ME-LIF as described by Fresta et al. [5]. As expected, Figure 3A shows that the stimulation of the macrophages with LPS + IFN-γ led to a decrease in the Arg peak and a corresponding increase in the Cit peak, confirming the conversion of Arg to Cit due to the activation of iNOS enzyme.

The Arg/Cit peak area ratio was calculated to be 2.93 and 0.25 for resting and stimulated macrophages, respectively. The presence of carnosine resulted in an Arg/Cit peak area ratio value of 0.29, very similar to that observed for cells stimulated in its absence (Figure 3B). This indicates that iNOS activity is not affected by the presence of this dipeptide, confirming our previous finding obtained by HPLC [86] and highlighting the suitability of ME-LIF to reinforce or be used in place of standard methods.

#### 2.5.2. Investigation of Carnosine Effect on NO Degradation by ME-EC

Very recently, we were able to demonstrate the peculiar ability of carnosine to increase the rate of NO degradation into its non-toxic end-products using a HPLC method, providing deeper insights into the carnosine-mediated transformation into NO_2_^−^ [139].

**Figure 4:**
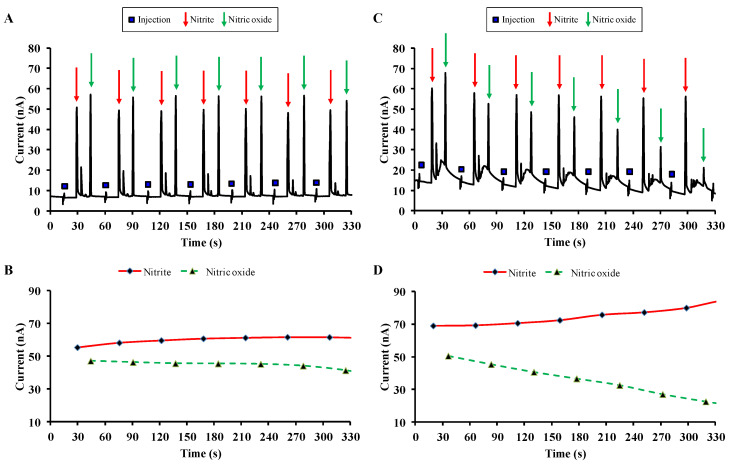
Detection of NO and NO_2_^−^ in a cell-free system using ME-EC. (**A**,**B**) show a representative electropherogram and the quantification (peaks’ height) of NO and NO_2_^−^ in a solution containing the NO donor DEA/NO in the absence of carnosine, while in (**C**,**D**) carnosine is present in the solution.

Here we show the application of ME-EC to study the effect of carnosine on the NO degradation in a cell-free system. The solution containing the NO donor diethylammonium (Z)-1-(N,N-diethylamino)diazen-1-ium-1,2-diolate (DEA/NO) in the absence or in the presence of carnosine were prepared according to the protocol describe elsewhere [86]. The fabrication of the PDMS-based microfluidic devices along with the details regarding platinum electrode fabrication, electrophoresis procedure, and electrochemical detection were previously described by Gunasekara et al. [85]. Figure 4 shows the representative electropherograms obtained analyzing a solution containing the NO donor alone (Figure 4A) or in the presence of carnosine (Figure 4C) by ME-EC.

Each electropherogram (Figure 4A,C) was obtained by multiple sequential injections from the same sample reservoir with a temporal resolution (separation time) of approximately 30 s and contains three different peaks: injection (blue square), NO_2_^−^ (red arrows), and NO (green arrows). As clearly shown in Figure 4B, reporting the curves for NO_2_^−^ and NO, obtained by measuring the peaks’ height, the concentration of both molecules in solution is quite stable during the entire electrophoretic run (330 s). On the other hand, the presence of carnosine led to an evident decrease of NO paralleled by an increase of NO_2_^−^ (Figure 4D). These results not only confirmed previous findings surrounding the role of carnosine in NO metabolism [86,139], but also provided additional information that could not be obtained from the HPLC method. Here, with ME-EC information was also obtained about NO, a diffusible and volatile compound with a half-life of 3–6 s in vivo, very difficult to identify and quantify in real-time.

## 3. Organs-, Organoids-on-a-Chip, and 3D Printing

The drug development process as well as the study of toxic effects coming from a specific drug treatment are taking advantage from the use of innovative systems, including organoids, mimicking the human physiological and/or pathological features [142]. Each year, the development of numerous drug candidates fails as a consequence of the adverse side effects in preclinical toxicology studies. Additionally, many of those selected to enter the clinical phase are terminated due to adverse events in humans. Therefore, the development of in vitro systems replicating the in vivo milieu and accurately predicting the safety of candidate compounds is strongly needed [143]. These systems will make it possible to avoid such failures, benefitting both patients and drug companies.

As previously mentioned, the shift from flat, 2D cell culture to 3D structures allows to mimic a more realistic biochemical and biomechanical microenvironments. In order to accomplish that transition, cells are grown inside chambers and channels to generate tissues or complete organs emulating their biology and integrative physiology. In 2004, Sin et al. developed a three-chamber microscale cell culture analog system on a one square inch silicon chip able to simulate the interactions between lung and liver [144], becoming a pioneer in the use of a microfluidic system to improve the understanding of organ–organ interaction [144,145]. In order to recreate the structural and functional features of human organs, the dynamic control of the cellular microenvironment was studied in a microfluidic device by creating different biomimetic models. This was later known as microfluidic organs-on-a-chip [146,147]. Within these devices, physiological functions such as fluid flow, cell–cell and cell–matrix interactions, size and shape parameters of the engineered organs are reproduced in a strictly controlled manner.

**Figure 5:**
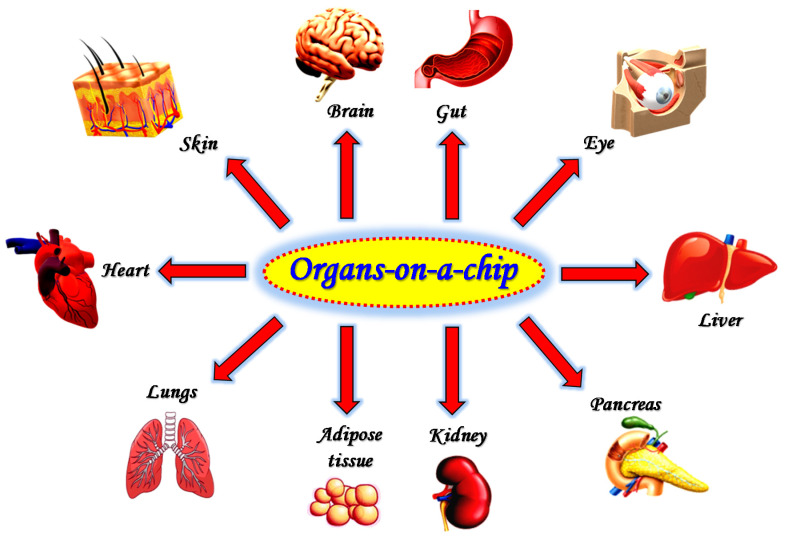
Schematic representation of microfluidic devices developed to reproduce the different organs/tissues of the body.

During the last decade, these findings have led to the massive efforts regarding the development of microfluidic organs-on-a-chip mimicking the entire human body [148,149]; in fact, at least one microfluidic-based model has been made to reproduce lung [147,150,151,152], gut [153,154,155,156,157], brain [158,159,160], eye [161,162], skin [163,164], liver [165,166,167,168], kidney [169,170], pancreas [171,172], adipose tissue [173,174], and heart [175,176,177] (Figure 5).

An overview of all these organs-on-a-chip is beyond the scope of the present review, but we want to focus on some examples related to their application to toxicology. In 2015, Brown et al. recreated the blood–brain barrier (BBB) structure on a chip, comprised of both a vascular chamber and a brain chamber separated by a porous membrane, allowing the cell-to-cell communication at neurovascular unit (endothelial cells, astrocytes, and pericytes) level, in order to mimic its physiology [159]. In this study, the authors measured the cell toxicity due to brain perfusion occlusion (50%) or cold shock (33 °C). They also demonstrated the possibility of growing human neurons in their system in such a way that the effects of drugs on neuronal survival and homeostasis could be evaluated in the context of the BBB, considering both drug permeability and its effect on the BBB itself. The lung is one of the most popular applications of organ-on-a-chip. Recently, Zhang and colleagues developed a novel 3D human lung-on-a-chip model, mimicking the organ-level structure and functions of the human lung, to evaluate the potential pulmonary toxicity due to the presence of TiO_2_ and ZnO nanoparticles [150]. Finally, Kim et al. developed a human gut-on-a-chip and demonstrated that the deregulated intestine bacteria proliferation and inflammatory phenomena are sustained by gut microbiome, inflammatory cells (by producing interleukin (IL)-8, IL-6, IL-1β, and tumor necrosis factor (TNF)-α), and peristaltic contractility [156]. One of the major strengths of their model is that each component can be varied independently, giving the opportunity to further improve this microsystem, for example by incorporating induced pluripotent stem cells (iPSCs) in order to obtain an ‘intestinal organoid’.

An organoid is a 3D multicellular in vitro tissue construct derived from stem cells (‘wild’ or iPSC), that mimics its corresponding in vivo organ [178]. The ability of pluripotent stem cells to differentiate in vitro in one of the three germ layers—endoderm (inner layer), ectoderm (outer layer), and mesoderm (middle layer)—combined with 3D cell culture methods satisfies the need to obtain reliable, realistic, and personalized in vitro models to investigate, in more detail, the development and dynamism of particularly complex organs, such as the CNS [179,180] or the mammary gland [181,182], or in specific context, such as tumorigenesis [183]. The organoid ‘body’ assumes an increasing complexity over the time, similar to what happens in physiological conditions—such as cell–matrix and cell–cell interactions, cell morphology, proliferation, and differentiation [183]—offering an improved understanding of the mechanisms underlying human organogenesis in complex diseases with specific genotypes and phenotypes that cannot generally be studied and/or reproduced in animal models.

The development of organs and organoids, including human iPSC derived ones, on microfluidic devices and the possibility of using separation science [184] including ME to address biochemical questions, can be of interest in the case of newly developed medical treatments for human diseases that present several limitations, such as individual differences among patients, outcomes prediction, and time-consuming drug testing, or for some pathologies where the animal models are still missing [185]. Additionally, these combined devices could represent a powerful tool to predict the possible teratogenic effects of drugs, an essential step in the preclinical development of drug candidates, since these studies currently rely on animal models that do not always adequately mimic human development [186].

Microfluidic techniques can provide a continuous, cyclic, or intermittent perfusion therefore creating the potential to overcome the ‘static nature’ that limits organoid 3D cultures. Pluripotent and adult stem cells can be grown in a compartment (chip) filled with matrigel and connected to a microfluidic system that allows the exchange of nutrients and/or fluids [187]. Inside the chip, these cells self-organize into organoids with well-defined features [16].

Two detailed reviews regarding the development and applications of microfluidic organoid-on-a-chip platforms [16,188] and a well addressed review on analytical strategies using separation science and microfluidic techniques were recently published [184]. Here we will describe some selected examples highlighting the numerous advantages offered by these innovative technologies.

Very recently, Shirure et al. developed a microfluidic device simulating the in vivo vascular component of the environment around a tumor [189]. First of all, this tumor organoid-on-a-chip demonstrated the ability to mimic perfusable blood vessels [190,191,192,193], thus representing an advantageous platform to overcome the problem of limited nutrient supply and life span in conventional organoid models. Different aspects related to tumor progression, such as cell proliferation and the formation of new blood vessels, were monitored by using their system. Thus, the in vivo tumor microenvironment features are recapitulated and observed in vitro. Additionally, the possibility to support vascularized tumoroids for 22 days made it possible to observe the toxicological effects caused by vascular perfusion with paclitaxel, suggesting the advantageous use of this combined platform as preclinical patient-derived model to analyze responses to chemotherapy. Overall, the above-mentioned organoid-on-a-chip technology represents a very innovative and stable (several weeks) example to reproduce and monitor both physiological and pathological changes of multiple entities and allowing for the test of drug activity (toxicity, safety, and efficacy).

Likewise, during natural organ development organoids need the activation of morphogenetic signaling pathways in order to self-organize [194]. Therefore, the addition of exogenous morphogens to the medium at specific time points is necessary. The morphogen diffusion and the release of cell-secreted soluble factors create a biochemical gradient in the stem cell niche [188,195]. The simulation of the critical morphogen distributions (similar to the organogenesis taking place in vivo) represents a limitation of 3D cultures. Recently, Demers et al. showed how the spatially self-organization and differentiation of neural tube composed by embryonic stem cells into motor neurons can be achieved through a combination of microengineering and microfluidic techniques [196]. Specifically, by monitoring the morphogen gradients with a microfluidic approach, it was possible to study the effects of different morphogen concentrations on the motor neuron differentiation of mouse embryonic stem cells, and ultimately choose the concentration able to mimic as much as possible the in vivo scenario.

The creation of biochemical gradients by using a microfluidic approach represent a good strategy for toxicological studies as well as for the deep investigation of the effects coming from the specific pharmacological treatment in structural components of the human body [197]. On this point, iPSCs-derived brain organoid was employed to model the pathological features (e.g., abnormal cortical development) occurring under prenatal environmental exposure to toxins such as nicotine [198]. In a separate application, Wang et al. build up a microfluidic system of BBB obtained by derivatizing brain microvascular endothelial cells from iPSCs that allows the investigation of both permeability and toxicity of drugs (caffeine, cimetidine, and doxorubicin) [199].

In this respect, it is worth mentioning that many research groups, following the pioneering biochemical engineering research carried out by Shuler group [144,200,201,202], are working on the development of different combinations of organs/organoids-on-a-chip in order to reach a higher level of complexity, even closer to the human body—named “body-on-a-chip” or “human-on-a-chip”—with the aim to accurately predict interactions between organs and tissues under physiological as well as pathological conditions [149,203].

Finally, the advent of 3D bioprinting has provided the ability to fabricate layer-by-layer 3D organized heterogeneous structures that are morphologically and physiologically similar to the relevant in vivo cytoarchitectures. This uses rapid prototyping and additive manufacturing technologies for depositing cell-laden bio-ink and has opened up new possibilities for organ and organoids ‘manufacturing’. Initially proposed by Mironov et al. in 2003 [204], 3D bioprinting approaches have quickly evolved, with the aim to support both the creation of specific in vitro experimental models and to fulfill the demand of innovative approaches in the field of regeneration of tissues/organs. The coupling of bioprinting with microfluidics technology for the engineering of 3D tissues and organs is widely used nowadays. This microfluidics-assisted bioprinting could provide a unique opportunity to create complex biological units by 3D bioprinting that are ‘microfluidically’ perfused and regulated [205]. Successful examples have been described that address both the fabrication of microfluidic devices using 3D printing [206,207,208] and for the bioprinting of 3D tissues/organs in combination with microfluidics [205], leading to the synergistic combination of two different technologies that allow the development of systems mimicking the in vivo environment [209] that can be extensively used for drug development [210,211,212].

## 4. Conclusions

During the last two decades, a plethora of combined systems coupling 2D and 3D cell culture experiments with microfluidics have been developed with the aim to enhance the predictive power of in vitro and in vivo models, and then allowing to replicate the in vivo milieu and accurately predict the safety profile of drug candidates. The use of these microfluidic-based devices offers numerous advantages including the fast separation and quantification of multiple chemical species, high sensitivity and reproducibility, the possibility to grow and manipulate single or group of cells, and to study heterogeneity of cell populations. It also makes it possible to investigate the simulation of cellular response mimicking the function of the human organism as well as study drug–organ interactions. Recent integration of microfluidics and 3D bioprinting technologies have further expanded the possibility of creating personalized and specific innovative in vitro models for different scenarios in biological and biomedical research. It is clear that microfluidics represents as a powerful tool for the study of cell biology and preclinical toxicology, and will play an essential role in drug discovery and development processes.
